# Cytoreductive Surgery and HIPEC for Peritoneal Carcinomatosis in the Elderly

**DOI:** 10.1155/2014/987475

**Published:** 2014-04-16

**Authors:** J. D. Spiliotis, E. Halkia, V. A. Boumis, D. T. Vassiliadou, A. Pagoulatou, E. Efstathiou

**Affiliations:** ^1^Department of Surgery, Metaxa Cancer Hospital, Piraeus, Botassi 51, 18537 Piraeus, Greece; ^2^Hellenic Society of Peritoneal Surface Malignancy, Botassi 51, 18537 Piraeus, Greece; ^3^Department of Anesthesiology, Metaxa Cancer Hospital, Piraeus, Greece

## Abstract

*Background*. The combined treatment of peritoneal carcinomatosis with cytoreductive surgery and hyperthermic intraperitoneal chemotherapy is a rigorous surgical treatment, most suitable for young and good performance status patients. We evaluated the outcomes of elderly patients undergoing CRS and HIPEC for peritoneal carcinomatosis with careful perioperative care. * Methods*. All consecutive patients 70 years of age or older who were treated for peritoneal carcinomatosis over the past five years were included. Primary outcomes were perioperative morbidity and mortality. Secondary outcomes were disease-free survival and overall survival. * Results*. From a pool of 100 patients, with a diagnosis of PC who underwent CRS and HIPEC in our center, we have included 30 patients at an age of 70 years or older and the results were compared to the patients younger than 70 years. The total morbidity rate was 50% versus 41.5% in the group younger than 70 years (NSS). The mortality rate was 3.3% in the elderly group versus 1.43% in the younger group (NSS). Median overall survival was 30 months in the older group versus 38 months in the younger group. * Conclusion*. Cytoreductive surgery and HIPEC for peritoneal carcinomatosis may be safely performed with acceptable morbidity in selected elderly patients.

## 1. Introduction

Peritoneal carcinomatosis (PC) is associated with a poor prognosis, and, once it is diagnosed, survival is generally less than 6 months [1, 2]. Peritoneal carcinomatosis represents a devastating form of cancer progression and the pathogenesis of this clinical entity can be explained by several biological models and a better understanding of underlying tumor kinetics and cellular dissemination mechanisms [3].

A considerable number of patients presenting with peritoneal carcinomatosis from digestive or gynecological cancers are aged 70 or older. Offering a safe and appropriate treatment to elderly patients with PC presents a challenge for healthcare resources. The combination of cytoreductive surgery (CRS) and hyperthermic intraperitoneal chemotherapy (HIPEC) plays an important therapeutic role in patients with PC [4]. On the other hand, elderly patients are traditionally associated more frequently with comorbidities and a reduced capacity to recover or tolerate aggressive surgery [5, 6]. Several recent results have shown that age alone does not influence the outcome of surgery and cancer-specific survival in these patients is similar to that of younger patients [7–9].

**Figure 1:**
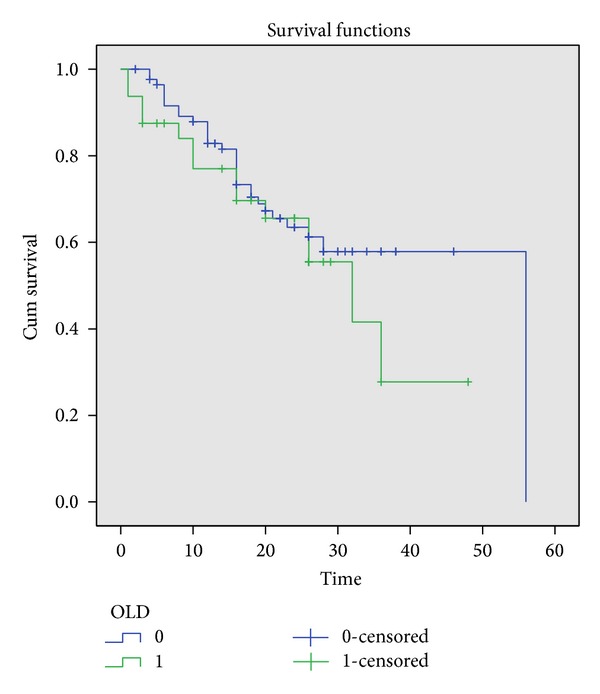


As for the management of PC nowadays, many patients are treated in specialized centers around the world with extensive CRS with peritonectomy procedures combined with HIPEC [2] and encouraging disease-free and overall survival results have been reported. In the past, in the few randomized trials that were performed, age was used as a selection criterion, investigating the outcomes in patients younger than 70 or 65 years [10, 11]. However, patients with peritoneal carcinomatosis stemming from any tumor site are 70 years old or older at the time of diagnosis [12]. The risks and benefits of CRS and HIPEC in elderly patients have not been clearly defined (Figure 1).

Using prospectively collected data from our institution with specialized interest in peritoneal surface malignancies, we examined the outcomes in the elderly patients who underwent CRS and HIPEC.

## 2. Patients and Methods

### 2.1. Patients

From a pool of 100 patients with a diagnosis of PC who underwent CRS and HIPEC in our center in Greece, in this study we have included patients at an age of 70 years or older and the results were compared to the patients younger than 70 years.

Data regarding patient characteristics, surgical procedures, perioperative outcomes, and survival outcomes were prospectively collected.

Regular followup was performed every three months for the first year and at six-month interval thereafter.

### 2.2. Preoperative Evaluation and Management

The eligibility for CRS and HIPEC procedures is decided after physical examination and double contrast-enhanced CT scans of the chest, abdomen, and pelvis. In addition, a positron emission tomography (PET) scan was performed to assess the extent of disease if necessary.

Each case was put to discussion at a multidisciplinary team meeting attended by surgical oncologists, medical oncologists, radiation oncologists, anaesthesiologists, cancer care nurses, and research staff.

In all patients a jugular or subclavian central venous catheter was placed and they received prophylactic antibiotic treatment with cefazolin 200 mg and metronidazole 500 mg every six hours during surgery and postoperatively.

Mechanical bowel preparation was performed on all patients.

### 2.3. Cytoreductive Surgery

In both institutions, procedures were performed by a specialized surgical team, led by the same surgeon (JS).

Patient position was supine; a midline laparotomy was performed, followed by assessment of the extent of disease with the use of the peritoneal cancer index (PCI) [13]. The PCI is a combined assessment of thickness of the lesion, size and distribution of tumor deposits in different abdominal regions, resulting in a numerical score which represents a quantification of the extent of the disease [13].

Cytoreductive surgery was performed using peritonectomy procedures, as described by Sugarbaker [14].

The macroscopic result of cytoreduction was assessed and recorded using the completeness of cytoreduction (CC) score [15].

### 2.4. HIPEC

After cytoreduction, HIPEC was performed by infusion of a heated chemoperfusate into the abdomen using either the Coliseum technique or the closed abdomen technique at approximately 42.5°C for 90 minutes.

The drug protocol that was used has been described previously by our team [16], with a 30% dose reduction in the aged group.

### 2.5. Statistical Analysis

Perioperative morbidity and mortality were the primary outcomes of this study.

Survival was evaluated by the Kaplan-Meier survival analysis.

Overall survival was defined as the time between the CRS and HIPEC and the date of death or last followup.

Disease-free survival was defined as the time between the CRS and HIPEC and the date of recurrence.

A *P* value of <0.05 was considered statistically significant.

## 3. Results

Between January 2007 and July 2011, 100 CRS and HIPEC procedures were performed. Thirty patients of 70 years or older (mean age 74.5 years) underwent combined treatment with CRS and HIPEC.

Details on concomitant disease and medical history are given in Table 1.

In all patients, peritoneal carcinomatosis occured and was discovered during followup for their primary tumor. Median time between the primary tumor resection and CRS + HIPEC was 18 months (6–180). In the older group, the mean PCI was 25 (4–39) and a complete removal of the peritoneal disease (CCS0) was achieved in 16 patients (53.3%), while in the younger group (<70 years) the mean PCI was 24 (3–39) and a CCS0 was achieved in 55.7%. Both differences are not statistically significant. All the data concerning the intraperitoneal procedures are presented in Table 2.

### 3.1. Morbidity and Mortality

In fifteen patients (50%) of the aged group, the CRS and HIPEC were uncomplicated. In the group under 70 years, in 41 patients (58.5%) no complications were observed. The mortality rate was 1/30 (3.3%) in the older group versus 1/70 (1.4%) in the younger group, respectively (NS).  Table 3 demonstrates the morbidity and mortality rates. Details regarding the complications and their management are presented in Table 4.

Three patients from the older group (10%) required surgical intervention in order to deal with acute and severe complications compared to five patients (7.1%) from the younger group.

### 3.2. Survival Outcomes

Table 5 demonstrates the overall survival rates between the two groups. Age plays a role in the overall survival rates three years after the initial operation.

Univariate analysis was performed to assess the influence on overall survival of the following factors: age > 75, histologic type of tumor (mucinous adenocarcinoma versus adenocarcinoma), peritoneal cancer index (PCI), completeness of cytoreduction score (CCS), amount of blood transfused, duration of CRS + HIPEC, and occurrence of postoperative complications.

Low PCI (<10), completeness of cytoreduction (CCS0), and tumor histology were the factors that influenced overall survival.

## 4. Discussion

Over the past 30 years the global burden of cancer has more than doubled, so the ageing population and the associated rise in cancer prevalence lead to an increase in demand of cancer treatment. Moreover, life expectancy has extended up to ages over 80 years. These factors together with the availability of modern surgical equipment, new antibiotic regimens, and the high standards of anesthetic and ICU care may reduce the risk of age as a preoperative selection criterion.

Studies from other centers confirm that CRS + HIPEC can be safely performed in elderly patients, including octogenarians [17, 18]. In the past, age has been regarded as a limiting factor for aggressive or curative therapies.

In the last decade, data in the surgical literature suggest a need for changing this notion. Reports have shown acceptable outcomes in terms of morbidity and mortality in patients older than 70 years of age undergoing gastrectomy for gastric cancer [19], pancreatectomy for pancreatic cancer [20], or liver metastasectomies [21].

Our study shows that CRS + HIPEC can be performed safely in patients aged 70 years or older with an acceptable morbidity and mortality, comparable to patients younger than 70 years.

Primary outcomes in our study were overall survival, which is 52% in five years for the younger group versus the older group, in which it is 30%. This result demonstrates that three years after the initial operation there is a survival benefit in the younger population.

To optimize the outcomes of elderly patients undergoing CRS + HIPEC it is important to avoid unnecessary splanchnic resections. In our study low PCI (<10), completeness of cytoreduction (CCS0), and tumor histology were the factors that influenced overall survival [18].

The study of Klaver et al. [18] demonstrates equal results concerning the morbidity and mortality in elderly colorectal patients undergoing CRS + HIPEC. The study presents the results in twenty-four patients aged >70 years, but they did not compare their results against adults <70 years.

Also Foster et al. [17] presented a retrospective analysis of twenty patients over the age of 65 who presented for consideration of CRS + HIPEC and suggested that CRS + HIPEC can be safely performed in elderly patients including octogenarians.

Our study is reporting the outcomes of CRS + HIPEC in two age groups.

Other studies in the past have evaluated the safety, effectiveness, and feasibility of other major abdominal operations in the elderly, such as liver, pancreatic, gastric, and ovarian cancer surgery, showing a low mortality and acceptable morbidity with a careful patient selection process [19–22].

The role of HIPEC in the management of peritoneal carcinomatosis worldwide remains unclear with excellent result in well selected patients and especially in those of colorectal and ovarian origin. Over the past ten years, randomized controlled trials have shown prolongation of survival with the combination treatment as compared with standard care of conventional surgery and systemic chemotherapy [23].

The studies in elderly population have a bias concerning the quality of life and the influence of this aggressive approach of treatment in the comorbidities and its role in long-term survival. This factor may play a role in the survival of aged patients in our study, who, after a three-year period, have statistically significant lower survival rates compared to the younger group.

Votanopoulos et al. recently reported a series of 81 patients older than 70 years who underwent cytoreductive surgery and HIPEC [24]. The 30-day mortality was 13.8% and severe postoperative complications occurred in 38% of the patients, results which are similar to ours.

In conclusion, more data are needed to assess the survival rates and to identify factors of influence on morbidity, mortality, and quality of life of patients receiving CRS + HIPEC.

Also the treatment is feasible in patients over 70 years with good performance status and is well selected after consideration of the tumor volume, grading, and the type of resection. These factors must be the inclusion criteria for elderly patients in clinical trials to determine the best results.

## Figures and Tables

**Table 1:** Patient characteristics (*n* = 30).

	*n*
Gender	
Male	9
Female	21
Age (years)	
70–75	21
75–80	8
>80	1
Medical history—concomitant disease	
None	4
Diabetes	11
Hypertension	16
Ischemic heart disease	8
Location of primary tumor	
Colorectal	11
Ovarian	13
Gastric	1
Pseudomyxoma	2
Sarcoma	2
Mesothelioma	1

**Table 2:** Comparison of intraoperative characteristics between the groups older and younger than 70 years.

	Group <70 yrs (*n*)	Group <70 yrs (%)	Group ≥70 yrs (*n*)	Group ≥70 yrs (%)
*n*	70		30	
PCI	24		25	
Completeness of cytoreduction				
CC-0	39	55.7	16	53.3
CC-1	24	34.2	7	23.3
CC-2	4	5.7	5	16.6
CC-3	3	4.2	2	6.6

**Table 3:** Morbidity and mortality results between the two groups.

	Group <70 yrs (*n*)	Group <70 yrs (%)	Group ≥70 yrs (*n*)	Group ≥70 yrs (%)	
Total morbidity rate	29/70	41.5	15/30	50	NS
Total mortality rate	1/70	1.4	1/30	3.3	NS

**Table 4:** Complications in both groups.

	Group <70 yrs (*n*)	Group ≥70 yrs (*n*)
Pulmonary embolism	2	4
Hypertension	6	8
Atrial fibrillation	5	5
Pneumonia	1	2
Leucopenia	4	7
Prolonged ileus	8	10
Urinary tract infections	4	6
Drainage of intra-abdominal collection	2	4
Reoperation	5	3
Pancreatic fistula	1	1
Hemorrhage	3	1
Peritonitis	1	1

**Table 5:** Survival rates between the two groups.

	Group <70 yrs (%)	Group ≥70 yrs (%)	
6 months	91.5	87	NS
12 months	82.8	75	NS
18 months	72	70	NS
24 months	63	65	NS
36 months	58	27.7	*P* < 0.05
48 months	58	27.7	*P* < 0.05

Maximum followup: 56 months.
